# Characterization of vascular tortuosity throughout the murine oxygen-induced retinopathy model of ischemic retinopathy

**DOI:** 10.1172/jci.insight.200679

**Published:** 2026-06-02

**Authors:** Kyle V. Marra, Tomoya Murakami, Jimmy S. Chen, Edith Aguilar, Jacob Robinson, Maxwell Prenner, Richard Daneman, Martin Friedlander, Eric Nudleman

**Affiliations:** 1 Shiley Eye Institute, Department of Ophthalmology, University of California, San Diego, San Diego, California, USA.; 2 Department of Ophthalmology, Faculty of Medicine, University of Tsukuba, Tsukuba, Ibaraki, Japan.; 3 Lowy Medical Research Institute, La Jolla, California, USA.; 4 Molecular Medicine, Scripps Research, San Diego, California, USA.; 5 Department of Pharmacology and Neurosciences, University of California, San Diego, La Jolla, California, USA.

**Keywords:** Ophthalmology, Vascular biology, Biomarkers, Mouse models, Retinopathy

## Abstract

Vascular tortuosity (VT) is a critical biomarker of disease progression that informs the decision to treat ischemic retinal disorders, particularly retinopathy of prematurity (ROP). The murine oxygen-induced retinopathy (OIR) model is the most widely used model of ischemic retinopathy. Although VT has been described in OIR, its temporal dynamics have not been systematically defined. In this study, a semiautomated artificial intelligence–based (AI-based) pipeline was used to quantify VT throughout OIR. Retinal flat mounts from age-matched normoxic and OIR mice (P10–P56) underwent vessel segmentation using a generative adversarial network (GAN), and VT was quantified as a cumulative tortuosity index with the iROP-Assist algorithm. Concurrently, standard OIR outcomes of neovascularization (NV) and vaso-obliteration (VO) were quantified using the algorithm at http://oirseg.org/. NV peaked at P17 and resolved by P23, while VO regressed over a similar interval. VT peaked with NV at P17 but remained elevated through P56. These temporal changes mirror both the development of VT and its persistence after NV regression observed clinically in ROP. Collectively, these findings establish VT as a durable, quantifiable phenotype in OIR and expand the model’s utility beyond neovascular endpoints, providing a translational platform for investigating VT pathogenesis and evaluating the effects of therapeutic agents on VT.

## Abstract 2

A new AI-based tool analyzing mouse models of ischemic retinal disease demonstrates that vascular tortuosity peaks with disease but persists long after recovery, offering a novel method to study and treat vascular disease.

## Graphical abstract

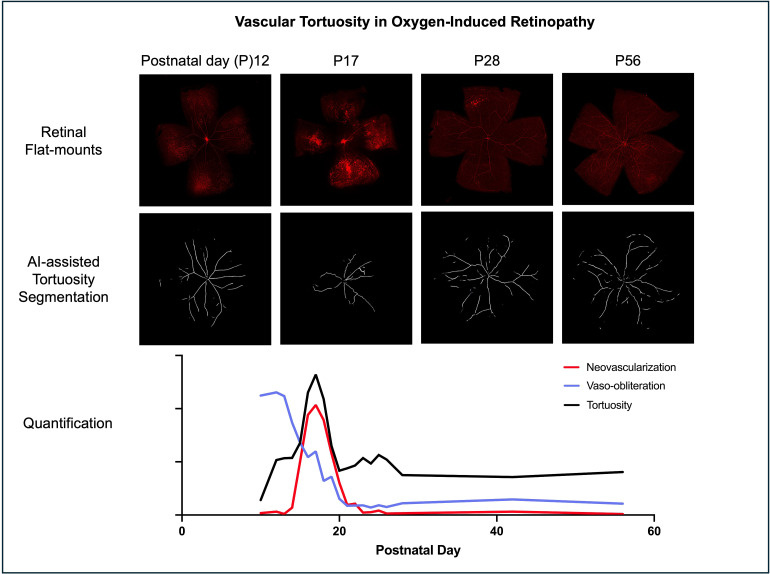

## Introduction

Vascular tortuosity (VT) is a clinically important biomarker of vascular disease. In retinopathy of prematurity (ROP), VT is a cardinal feature of disease severity and a critical determinant of treatment necessity (1, 2). Beyond the eye, increased VT is associated with systemic vascular pathology, including ischemic stroke, cardiovascular disease, and chronic kidney disease, underscoring its potential as a global biomarker of vascular dysfunction (3–9). Despite this clinical significance, an in vivo model of VT has not been rigorously characterized, limiting our ability to study its pathogenesis or investigate the effects of therapeutic strategies on VT.

The murine oxygen-induced retinopathy (OIR) model is among the most widely used preclinical models for evaluating ischemic retinal disease. OIR mice recapitulate key features of ischemic retinopathy — ischemia followed by neovascularization (NV) — and associated sequelae, such as vascular leakage and proliferation (10, 11). In the classic paradigm, newborn mice are exposed to hyperoxia from P7 to P12, inducing vaso-obliteration (VO) in the central retina. Upon return to room air on P12, the retina becomes acutely ischemic, driving a neovascular phase that peaks at P17. Historically, outcome assessments in OIR have focused on NV and VO as primary endpoints, with P17 as the canonical time point for measurement (10). In clinical ischemic retinopathies such as ROP, VT is central to diagnosis and management (1, 2), and it often persists long after NV has regressed (12). However, VT has not been systematically characterized in OIR mice. The current work aims to fill this gap in knowledge by quantifying VT throughout OIR to investigate the translational relevance between this experimental model and human disease.

Recently, we developed an artificial intelligence–based (AI-based) tool to automate the segmentation of major blood vessels from retinal flat mount images and quantify VT in OIR (13). VT was found to rise concurrently with NV in OIR (14). In the present study, this platform was leveraged to comprehensively characterize the temporal dynamics of VT in the OIR model versus normoxic (NOX) controls from P10 through P56. Our findings establish VT as a quantifiable and durable phenotype in OIR, expanding the utility of this model for studying ischemia-driven vascular remodeling and for evaluating the effects of therapeutics on VT.

All retinal flat mounts are publicly available (https://figshare.com/articles/dataset/NOX_vs_SOIR_Characterization_over_Time_zip/29848754?file=56997977) and may be used in addition to our previously published open source dataset (dx.doi.org/10.6084/m9.figshare.23690973) (15) to independently discover, validate, and characterize novel biomarkers or to improve the quality of current AI algorithms.

## Results

### Characterization of NV, VO, and VT throughout OIR.

**Figure 1:**
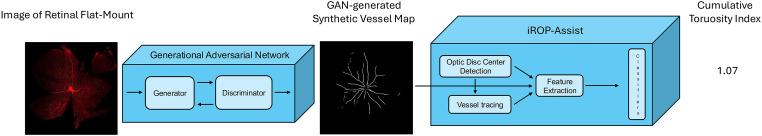
AI-based calculation of vascular tortuosity. In our previous publication (14), flat-mounted images were manually segmented by 4 graders, cross-validated, and used to train a GAN to automatically segment large blood vessels. GAN-generated synthetic vessel maps were input into the iROP-Assist algorithm that calculated the cumulative tortuosity index, a previously validated (14) measure of vascular tortuosity on both fundus images of infants with ROP as well as OIR mice.

**Figure 2:**
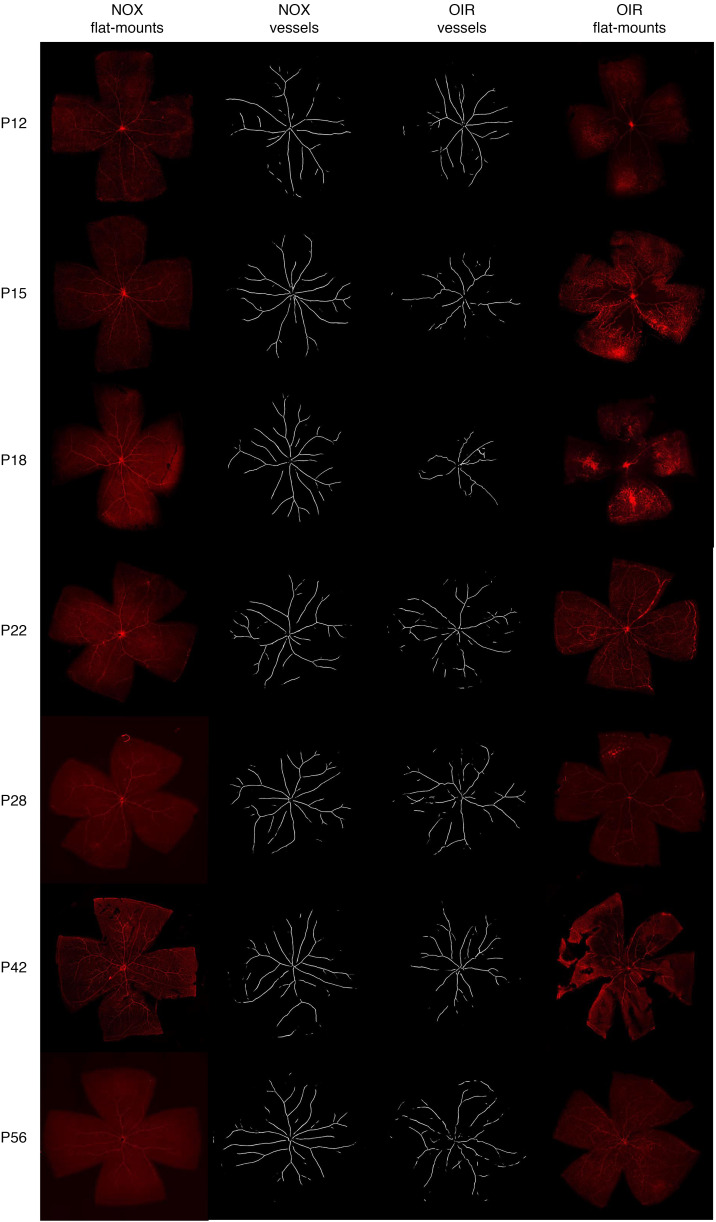
Representative images of NOX and OIR flat mounts with AI-generated vessel segmentation maps. Retinal flat mounts of NOX and OIR mice with vascular tortuosity closest to the mean VT were selected as representative images of time points in OIR. Synthetic images for each retinal flat mount were generated for qualitative side-by-side comparison of VT between NOX and OIR retinas. Original magnification, x10.

Age-matched NOX and OIR mice were sacrificed and retinal flat mounts were prepared at P10, daily from P12 to P25, and on P28, P42, and P56. NV and VO were quantified using the publicly available deep learning–based algorithm available at http://oirseg.org/ (16), while VT was quantified using our previously published pipeline (13, 14). Segmentation maps of the superficial vasculature of retinal flat mounts were output by a generative adversarial network (GAN), and these synthetic vessel maps were then input into iROP-Assist, an algorithm previously validated on human fundus images from infants with ROP (17), in order to calculate a cumulative tortuosity index (CTI) that quantifies VT (Figure 1). Representative images of NOX and OIR flat mounts at pathophysiologically relevant time points within and beyond the classic OIR model are provided in Figure 2.

**Figure 3:**
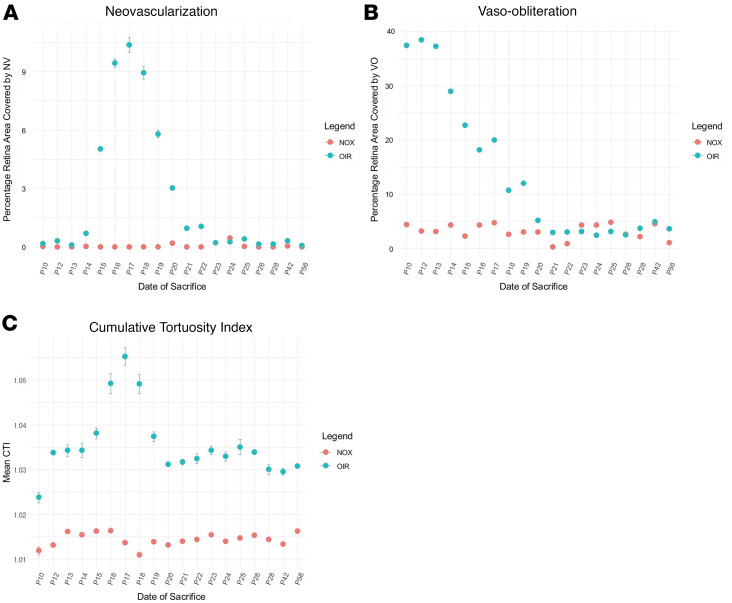
Quantification of neovascularization, vaso-obliteration, and vascular tortuosity in OIR and age-matched NOX mice. Mice were sacrificed on P10, daily from P12 to P26, and on P28, P42, and P56. Retinal flat mounts were quantified for (**A**) NV, (**B**) VO, and (**C**) VT. NV peaked at P17 in OIR mice and regressed by P23. VO had developed at P10 in OIR mice and decreased until reaching levels comparable to those of NOX mice at P23. Relative to NOX mice, OIR mice exhibited increased CTI at P10 that peaked at P17. CTI subsequently regressed in OIR mice but remained elevated compared with levels observed in NOX mice, even at P56.

NV, VO, and VT in NOX and OIR mice were quantified and compared at each time point (Figure 3). In OIR mice, NV appeared at P14, peaked at P17, and regressed by P23. NV was statistically significantly greater in OIR mice relative to that in NOX controls from P14 to P26 at all time points except P23 and P24 (Supplemental Table 1; supplemental material available online with this article; https://doi.org/10.1172/jci.insight.200679DS1). There was no NV in NOX mice. VO developed by P10 in OIR mice and decreased until reaching levels comparable to NOX controls at P23. VO was statistically significantly greater in OIR mice relative to that in NOX controls from P10 to P25 at all time points except for P20 and P23 (Supplemental Table 2). Small VO values in NOX mice were quantified by http://oirseg.org/; however, this reflects inclusion of the optic nerve head by the algorithm during VO quantification. On manual review of retinal flat mounts, no VO was present in NOX mice. These results corroborated previous reports characterizing NV and VO throughout the OIR model (10). Relative to NOX mice, OIR mice exhibited increased CTI at P12 (*P* < 0.001). Analogously to NV, VT peaked at P17. VT subsequently regressed in OIR mice but remained significantly elevated compared with levels observed in NOX mice at time points as late as P56 (Supplemental Table 3). The number of mice used for each condition (NOX and OIR) at each time point is provided in Supplemental Table 4.

### Interlaboratory batch effects of NV, VO, and VT measurements.

Retinal flat mounts were prepared and imaged from 2 independent laboratories, necessitating the evaluation of possible laboratory-dependent batch effects on NV, VO, and CTI. To investigate batch effects, these measurements were compared between laboratories for retinas prepared at the same postnatal age. The number of retinas used from each laboratory for each condition is provided in Supplemental Table 5.

Linear models were used to evaluate the effects of laboratory, postnatal age (categorical), and experimental condition (OIR vs. NOX). Main laboratory effects and laboratory-by-age interactions were investigated (Supplemental Table 6). The main laboratory effect metrics investigate whether, on average across all ages and across both NOX and OIR conditions, the measurements from 1 laboratory were significantly higher or lower than those obtained from the other laboratory. The main lab effects for NV, VO, and CTI were *P* = 0.433, *P* = 0.584, and *P* = 0.768, respectively, indicating no evidence of a consistent systematic offset between the 2 laboratories for any outcome. The laboratory-by-age interaction tests whether differences between laboratories are dependent on the postnatal age at which the measurements were taken. The laboratory-by-age interactions were statistically significant for NV and VO (both *P* < 0.001), indicating that laboratory-associated differences in these metrics were not constant across postnatal ages but instead varied in an age-dependent manner. In contrast, the laboratory-by-age interaction for CTI was not significant (*P* = 0.484), which demonstrated consistent agreement between data from laboratories across all time points. Metric distributions stratified by laboratory for NOX and OIR mice demonstrate substantial overlap between laboratories at most ages, with divergence at select developmental stages for NV and VO but not for CTI (Supplemental Figure 1).

Altogether, these data suggest no significant overall interlaboratory variability for NV, VO, and CTI and that, while NV and VO exhibit modest age-specific laboratory variability, large-vessel tortuosity measurements quantified by CTI remain a stable and reproducible metric across experimental sites as well as postnatal time points.

Age-related relationships between CTI and the conventional OIR outcome measures of NV and VO were further assessed using Spearman’s rank correlation analysis at each postnatal age (Supplemental Table 7). CTI demonstrated weak or nonsignificant correlations to both NV and VO at most postnatal ages, indicating that VT was largely dissociable from NV and VO. A moderate positive association between CTI and NV was observed at the canonical peak of disease activity (P17; *r* = 0.42, *P* = 0.049), which coincides with maximal NV and maximal tortuosity. Another significant positive association between CTI and NV was found at P56 (*r* = 0.52, *P* = 0.004), when NV had largely regressed in all animals, but increased CTI persisted. CTI exhibited minimal correlation with VO across most time points (*r* ≈ −0.1 to 0.3). A single significant negative association was detected at P16 (*r* = −0.47, p = 0.037).

## Discussion

Using a previously published semiautomated AI-based pipeline, we demonstrate that VT is elevated throughout the full-time course of the OIR model (13, 14). VT was increased during the vaso-obliterative phase (P10), peaked concurrently with NV at P17 and — unlike NV — remained significantly elevated through at least P56. This persistent elevation establishes VT as a durable phenotype in OIR, in contrast to the transient behavior of NV and VO, which both regressed back to levels comparable to NOX controls by P23.

The temporal course of vascular changes observed in OIR closely parallels clinical observations in ROP. Infants with ROP are born with incompletely vascularized retinas, are exposed to transient hyperoxia, and subsequently exhibit ischemia-driven NV that may regress spontaneously or with treatment (18). In the majority of cases, tortuosity persists even after NV resolution (19, 20). These similarities indicate that the OIR model recapitulates not only the onset and peak of VT seen acutely in ROP, but also its chronic persistence — an aspect not previously recognized in this preclinical model. Clinically, VT represents ongoing vascular dysfunction and influences long-term visual outcomes in ROP. To our knowledge, OIR represents the only preclinical in vivo model established to systematically study VT, underscoring its translational relevance.

The mechanisms that drive increased VT in ROP (i.e., plus disease) remain incompletely understood. One proposed hypothesis is that peripheral avascularity reduces the functional capillary bed, thereby lowering distal capillary resistance and promoting arteriovenous shunting (21–23). These low-resistance conduits bypass the normal capillary network, allowing larger volumes of blood to flow more rapidly through posterior arterioles and venules. The resulting increase in local shear stress and intraluminal pressure can cause vessel dilation and structural remodeling that manifest as VT. This theory is consistent with longstanding clinical observations that the caliber and configuration of posterior pole vessels strongly correlate with the severity of peripheral ROP (22–24). However, direct measurements of orbital or central retinal arterial flow by color Doppler imaging have not consistently demonstrated a global increase in peak systolic velocity in eyes with plus disease, suggesting that hemodynamic changes may be segmental, spatially heterogeneous, or occur distal to the optic nerve head, rather than reflecting a global increase in retinal blood flow (12). A complementary hypothesis is that ischemia-driven VEGF signaling acts directly on established posterior retinal blood vessels to promote morphologic remodeling (2), including endothelial cell proliferation (25). In a rat model of severe ROP, intravitreal injection of anti-VEGF decreased VT and altered endothelial cell division orientation in favor of elongation, supporting a mechanistic link between VEGF signaling and the development of plus-like VT (26).

Ischemia-driven endothelial cell proliferation may serve as a central mechanism underlying VT development and persistence (25, 27). In ischemic or neovascular conditions, altered hemodynamic forces and VEGF-mediated endothelial proliferation lead to excess endothelial cells within a constrained vascular pathway, resulting in tortuous vessel geometry. Once established, this tortuous configuration may be maintained through ECM and vascular basement membrane remodeling, which confers structural “shape memory” to the vasculature. The vascular basement membrane — composed of laminins, collagen IV, nidogen, and heparan sulfate proteoglycans — provides mechanical support to endothelial and mural cells, and durable alterations in matrix composition or organization can preserve abnormal vessel geometry long after the acute angiogenic stimulus resolves (28).

Future studies using the OIR model may investigate the pathophysiology of VT to help clarify these dynamics. Beyond its relevance to ophthalmology (3), VT is recognized as a meaningful biomarker across vascular beds, such as in ischemic heart disease, ischemic stroke, and chronic kidney disease (6–9). Preclinical measurements of VT in response to therapeutic interventions may provide useful insights into treatment effects on vascular health. OIR may therefore not only serve as a model for the pathogenesis of VT following ischemia/perfusion injury, but also as a system to evaluate acute and chronic effects of therapeutic interventions on VT. Accordingly, changes in CTI after treatment may reflect biologically meaningful vascular remodeling that warrants deeper mechanistic investigation.

An important feature of translational biomarkers is their reproducibility across experimental settings. In the current study, OIR mice and retinal flat mounts were prepared using 2 independent animal facilities, model system equipment, and laboratory settings with two different staining protocols and image acquisition microscopes. To investigate the generalizability of outcome metrics in OIR, the effect of laboratory-associated variability on NV, VO, and CTI was systematically assessed.

The lesion-based metrics of NV and VO demonstrated modest differences between laboratories depending on the postnatal age at which the measurements were taken, but there were no significant overall laboratory effects for these measurements. CTI demonstrated neither age-dependent interactions between laboratories nor an overall laboratory effect. The modest age-dependent laboratory variability observed for NV and VO may be a result of the technical sensitivity of lesion segmentation. Subtle differences in staining intensity or flat-mount geometry, as well as thresholding during automated segmentation, may have influenced area-based quantification of NV and VO across batches. Of note, time points that were split between labs often consisted of a single litter from each laboratory. These results may therefore be confounded by litter-related batch effects rather than age-related laboratory effects. In contrast, CTI was calculated based on segmentation of large superficial vessels and was normalized by vessel length, making the measurement less susceptible to local variation in signal intensity or background fluorescence. This methodological distinction may partially explain the superior cross-laboratory stability of CTI versus NV or VO.

The laboratory independence of CTI strengthens its value as a generalizable vascular phenotype and as a novel, durable candidate endpoint for future interventional studies. This cross-laboratory stability supports the use of CTI for VT quantification in multicenter studies and facilitates retrospective integration of datasets generated under heterogeneous experimental conditions.

Another fundamental question in identifying VT as a biomarker is whether it reflects the extent of NV or ischemic injury or, instead, captures additional dimensions of vascular pathology not fully represented by these conventional metrics. Age-specific correlation analyses demonstrated a moderate positive association between CTI and NV at the peak of the neovascular phase (P17), when both NV and VT are maximal, suggesting that the processes underlying pathologic NV and VT overlap during the acute phase of disease. This interpretation is consistent with shared upstream biological drivers, including hypoxia, VEGF signaling, and altered hemodynamic forces. At other postnatal time points, however, associations between CTI and NV were weak or nonsignificant, indicating that the coupling between these phenotypes is temporally restricted rather than persistent.

CTI and VO exhibited weak correlation coefficients at most ages, with only a single negative correlation observed at P16. Importantly, VO precedes both the development of VT and NV, and the phenotypes evolve on distinct timescales — VO peaks early at P10–P13 and regresses by P25, whereas CTI and NV peak later at P17, with CTI persisting well beyond this time point. Given their distinct temporal trajectories, strong cross-sectional correlations between VO and CTI would not be expected.

Collectively, these findings suggest that while VT shares upstream biological drivers with NV, it also reflects an independent aspect of vascular remodeling that extends beyond angiogenesis and ischemic capillary loss. A limitation of these analyses is that correlations were assessed cross-sectionally at each postnatal age rather than longitudinally within individual animals. As a result, these data cannot definitively establish causal or temporal relationships between CTI, NV, and VO within individual animals.

There are several other limitations to this study. First, quantification of VT was achieved with AI-based tools that are reliant upon image quality for accurate vessel segmentation. While in our previous studies the GAN utilized for vessel segmentation was found to accurately produce vessel maps comparable to those traced manually, errors inherent to flat mounting retinas (uneven staining, low signal-to-noise fluorescence, tissue distortions, pooled staining, leaflet curvature, etc.) may translate into poor AI-assisted segmentation. To mitigate this effect, all flat mounts and GAN-generated vessel segmentation images were manually reviewed prior to analysis with iROP-Assist, and images with obvious errors were excluded. Second, VT was calculated by iROP-Assist as the CTI of GAN-generated segmentations of major superficial retinal vasculature. This approach enables objective, reproducible quantification while reducing sensitivity to staining variability, but it does not fully capture more subtle changes in vessel caliber, 3-dimensional geometry, and small superficial capillary beds. Third, segmentation discontinuities can influence tortuosity quantification. Such discontinuities are more prevalent in peripheral vasculature, however, and GAN-generated vessel maps produced segmentations of more centrally located major superficial blood vessels emanating from the optic disc. Furthermore, the CTI metric is weighted toward continuous vessels and normalized by vessel length, thus limiting the effect of these artifacts. Future work incorporating synthetic distortion testing, perturbation analysis, or quantification of small capillary vessels could improve the robustness of the current AI-based tortuosity quantification tool.

In summary, this study establishes VT as a quantifiable, durable, and clinically relevant phenotype in the OIR model. Leveraging semiautomated AI-based analysis, this work shows that VT peaks with NV and persists long beyond its resolution, mirroring the clinical course of human ROP. These findings suggest that in addition to modeling ischemia-driven NV, OIR is a platform for studying VT and its response to therapeutic agents. Given VT’s recognized role as a biomarker in systemic vascular diseases, OIR mice may serve as a translational model for understanding VT across organ systems. The reproducibility of CTI further supports the applications of this AI-based quantification tool to large-scale, multicenter preclinical studies as well as to datasets generated under heterogeneous experimental conditions.

## Methods

### Sex as a biological variable.

Both male and female animals were used for NOX and OIR conditions. Previous studies have demonstrated no significant differences in the extent of retinopathy between male and female OIR mice (29).

### Animals.

Age-matched C57BL/6 mice (The Jackson Laboratory) were subjected to either the OIR model or NOX conditions as previously described (10). In OIR, neonatal mice were placed in a hyperoxic chamber (Bio-Spherix) on P7 for 5 days. On P12, mice were returned to ambient room air. Mice were sacrificed on P10, daily from P12–P25, and on P28, P42, and P56 (Supplemental Table 4). Eyes were enucleated, and the retinas were prepared as flat mounts for imaging using methods described previously (10). Retinal flat mounts were prepared in 2 laboratories. In the Friedlander lab, retinas were isolated using fine brushes to separate neural retina from underlying choroid and sclera. Fixation of retinal tissue was achieved using ice-cold 4% paraformaldehyde for 1 hour. Staining was performed overnight in PBS containing calcium and magnesium supplemented with 10 μg/mL of *Griffonia simplicifolia* isolectin B4 (GS-IB4, I21412, Thermo Fisher Scientific) to label vasculature. Retinas were cut into 4 leaflets and then flat mounted using SlowFade Gold Antifade Mountant medium (Thermo Fisher Scientific, S36937) or Antifade Mounting Medium with DAPI (Vector Laboratories, H-1800) mounting medium. Age-matched control mice were reared in NOX conditions and sacrificed at the same time points as OIR mice, and retinal flat mounts were prepared as above. Images were acquired via confocal microscopy using a Zeiss LSM 710 laser scanning microscope controlled by ZEN 2010 software (Zeiss), utilizing ×10 objective magnification and a tiled scanning configuration (5 × 5 tiles). In the Nudleman lab, enucleated eyes were fixed with ice-cold 4% paraformaldehyde for 1 hour, after which the retinas were dissected. The retinas were then blocked overnight at 4°C in a blocking buffer (0.5% bovine serum albumin, 10% NGS, and 0.5% Triton X-100 in PBS). Following blocking, some retinas were incubated with rat anti-mouse CD31 (1:200, BD Pharmingen, catalog 553370) in a blocking buffer for 2 days at 4°C with gentle rocking. This was followed by 4 washes in PBS and a subsequent 2-day incubation with goat anti-rat Alexa Fluor 568 (1:1,000, Thermo Fisher Scientific, A-11077). Other retinas were incubated directly with fluorescein-conjugated *Bandeiraea (Griffonia) simplicifolia* lectin-I (1:1,000, Vector Laboratories, FL-1101) in a blocking buffer for 1 day at 4°C with gentle rocking. After the final incubation, all retinas were washed four more times in PBS. They were then cut into 4 radial leaflets and flat mounted onto slides using Antifade Mounting Medium with DAPI (Vector Laboratories, H-1800). Images were acquired using a Zeiss Axioimager equipped with a monochromatic AxioCam 712 and controlled by Zen software. A ×10 objective was used to capture tiled scans of each retina.

### Vessel segmentation using a GAN.

Segmentation of superficial retinal vasculature was achieved using a GAN as previously described (13). Briefly, in our prior research, manual segmentations were used to train a GAN using Pix2PixHD software to generate high-resolution synthetic images of segmented superficial retinal vasculature (30). Pix2PixHD was trained in Python26 using a Nvidia K80 GPU (31). Segmentations generated using Pix2PixHD have comparable segmentation accuracy to U-Nets (30), which are convolutional neural networks designed for segmentation, as well as utility in generating vessel segmentations from fundus images (32). Optimization of the GAN was performed using the Adam optimizer at a learning rate of 10^-6^ for 100 iterations, followed by 100 additional iterations at a learning rate linearly decaying to 0. All retinal flat mounts from OIR and NOX mice were input into this GAN to generate vessel segmentation maps. Generated images were reviewed for error (such as missing vasculature, inaccurate segmentation, etc.) prior to inclusion in tortuosity quantification and statistical analysis.

### Quantification of NV, VO, and VT.

The percentage retinal area occupied by NV and VO was quantified using our published deep-learning algorithm, available at http://oirseg.org/ (16). VT was calculated as the CTI using AI-based tools as previously described (13, 14). GAN-generated vessel maps were input into a computer-based image analysis algorithm, iROP-Assist (17), for calculation of the CTI. The iROP-Assist algorithm was originally published as an algorithm for calculating plus disease on fundus images of infants with ROP. This algorithm quantifies VT in segmented images using the associated coordinates of the optic disc center. Pixels representing vasculature were extracted to create graphs of vessel segments using methods previously described by Ataer-Cansizoglu et al. (33) and definitions as described by Bolón-Canedo et al. (34) and Han. (35) For each image, we extracted CTI, which is calculated as the mean cumulative sums of angles between segmented vessels normalized by vessel length. While iROP-Assist was originally developed for human fundus images, the algorithm calculates tortuosity indices by quantifying angular deviation along vessel centerlines normalized to vessel length, making it agnostic to species-specific anatomy such as the presence or absence of vascular arcades. Of these tortuosity indices, CTI was previously demonstrated to be most appropriate for calculation of VT in OIR (14) and was again used as a quantitative measure of VT in the current study.

### Statistics.

Statistical analysis was performed using R 4.5.0. Two-sided Mann-Whitney *U* tests were used to compare quantitative measurements of CTI, NV, and VO between NOX and OIR mice at each postnatal age. To evaluate potential interlaboratory batch effects, NV, VO, and CTI were analyzed using linear models, including laboratory (categorical), postnatal age (categorical), and experimental condition (OIR versus NOX). Two model-based quantities were analyzed for each outcome (NV, VO, and CTI): (a) the main laboratory effect, representing the average difference between laboratories across all postnatal ages and experimental conditions, and (b) the laboratory-by-age interaction, which tests whether laboratory-associated differences vary by postnatal age. Spearman’s rank correlation was used to assess relationships between CTI and NV and between CTI and VO at each postnatal age in OIR mice. *P* < 0.05 was considered statistically significant.

### Study approval.

Experimental procedures using animals were approved by the Animal Care and Use Committees at The Scripps Research Institute and UCSD. Experiments were performed in accordance with the NIH *Guide for the Care and Use of Laboratory Animals* (National Academies Press, 2011). Protocols were approved by the Institutional Animal Care and Use Committees at The Scripps Research Institute and Scripps Memorial Hospital, La Jolla, California, USA, as well as UCSD.

### Data availability.

The raw data supporting the findings of this study are provided in the Supporting Data Values file accompanying this article. All retinal flat mounts are publicly available at https://figshare.com/articles/dataset/NOX_vs_SOIR_Characterization_over_Time_zip/29848754?file=56997977

## Author contributions

KVM and TM designed and performed experiments and wrote and edited the manuscript. JSC performed data analysis, figure generation, and manuscript editing. EA and MP assisted in performing experiments. JR assisted in maintaining animal colonies. RD, MF, and EN supervised the work and reviewed and edited the manuscript.

## Conflict of interest

The authors have declared that no conflict of interest exists.

## Funding support

This work is the result of NIH funding, in whole or in part, and is subject to the NIH Public Access Policy. Through acceptance of this federal funding, the NIH has been given a right to make the work publicly available in PubMed Central.

- VitreoRetinal Surgery Research Award to KVM and JSC.
- Heed Ophthalmic Foundation to JSC.
- National Eye Institute of the NIH, 1R56EY032513-01A1, to EN.
- Lowy Medical Research Institute grant to MF.

## Supplementary Material

Supplemental data

Supporting data values

## Version 1 06/02/2026

In-Press Preview

## Version 2 07/22/2026

Electronic publication
