# Supplementary material for: Characterization of vascular tortuosity throughout the murine oxygen-induced retinopathy model of ischemic retinopathy
Source: JCI Insight. 2026 Jun 2;11(14):e200679. doi: 10.1172/jci.insight.200679 (PMC13461169; doi:10.1172/jci.insight.200679)
Supplement: Supplementary file 1 [file jciinsight-11-200679-s392.pdf]

SUPPLEMENTAL DATA

	Normoxic Mice		OIR Mice		
**Age**	**Mean NV (%)**	**NV SD (%)**	**Mean NV (%)**	**NV SD (%)**	**P value**
P10	0.03	0.07	0.18	0.45	0.392
P12	0.01	0.03	0.32	0.97	0.026
P13	0.00	0.00	0.11	0.48	0.185
P14	0.03	0.12	0.70	1.20	0.002
P15	0.00	0.00	5.03	2.43	< 0.001
P16	0.01	0.03	9.43	4.32	< 0.001
P17	0.00	0.00	10.34	8.68	< 0.001
P18	0.01	0.04	8.93	3.85	< 0.001
P19	0.00	0.00	5.80	4.25	< 0.001
P20	0.20	0.24	3.03	3.10	0.01
P21	0.02	0.05	0.96	1.03	< 0.001
P22	0.00	0.00	1.07	1.50	< 0.001
P23	0.23	0.36	0.23	0.37	0.596
P24	0.47	0.59	0.27	0.61	0.357
P25	0.02	0.14	0.42	0.33	< 0.001
P26	0.00	0.00	0.15	0.27	0.02
P28	0.01	0.03	0.16	0.45	0.062
P42	0.06	0.15	0.31	0.81	0.01
P56	0.00	0.00	0.09	0.24	0.155

Supplementary Table 1: Quantification of retinal neovascularization in normoxic and oxygen-induced retinopathy mice. The percentage of retinal area occupied by neovascularization (NV) is reported as mean ± standard deviation (SD) for normoxic (NOX) and oxygen-induced retinopathy (OIR) mice at each postnatal age (P10–P56). NV was quantified using the fully automated deep-learning–based OIRseg algorithm. P values were calculated using two-sided Mann–Whitney U tests comparing NOX and OIR groups at each time point. These data demonstrate the emergence of pathological NV peaking at P17, and regression to near-baseline levels by approximately P23, consistent with the canonical temporal profile of the OIR model.

	Normoxic Mice		OIR Mice		
Age	Mean VO (%)	VO SD (%)	Mean VO (%)	VO SD (%)	P value
P10	4.33	0.99	37.44	7.22	< 0.001
P12	3.13	2.40	38.44	13.95	< 0.001
P13	3.09	1.74	37.26	5.24	< 0.001
P14	4.26	2.73	28.99	8.24	< 0.001
P15	2.18	1.15	22.71	1.68	< 0.001
P16	4.23	3.32	18.13	3.73	< 0.001
P17	4.69	2.67	19.93	8.10	< 0.001
P18	2.54	1.56	10.68	4.92	< 0.001
P19	2.96	1.98	12.02	6.07	< 0.001
P20	2.95	2.55	5.09	4.14	0.214
P21	0.21	0.28	2.89	1.71	< 0.001
P22	0.85	0.75	2.96	1.89	< 0.001
P23	4.22	2.33	3.04	2.15	0.128
P24	4.26	2.67	2.36	1.97	0.028
P25	4.79	2.55	3.05	1.31	0.027
P26	2.51	1.71	2.50	1.97	0.777
P28	2.14	1.68	3.70	4.45	0.467
P42	4.54	3.58	4.89	5.72	1
P56	0.98	1.25	3.57	3.63	0.021

Supplementary Table 2: Quantification of vaso-obliteration in normoxic and oxygen-induced retinopathy mice. The percentage of retinal area classified as vaso-obliterated (VO) is shown as mean ± SD for NOX and OIR mice at each postnatal age (P10–P56), as determined by automated segmentation with OIRseg. P values were calculated using two-sided Mann–Whitney U tests at each time point. VO is present during the hyperoxic phase at P12 and reaches a maximum during the early ischemic phase (P12-P13), then progressively decreases, approaching normoxic levels by P23–P26. Of note, non-zero VO values in NOX animals reflect erroneous inclusion of the optic nerve head region by the OIRseg algorithm rather than true areas of vaso-obliteration.

	Normoxic Mice		OIR Mice		
Age	Mean CTI	CTI SD	Mean CTI	CTI SD	P value
P10	1.012	0.007	1.024	0.020	0.124
P12	1.013	0.006	1.034	0.018	< 0.001
P13	1.016	0.006	1.034	0.028	< 0.001
P14	1.015	0.005	1.034	0.033	0.003
P15	1.016	0.005	1.038	0.022	< 0.001
P16	1.016	0.006	1.051	0.046	< 0.001
P17	1.014	0.005	1.055	0.044	< 0.001
P18	1.011	0.007	1.049	0.024	< 0.001
P19	1.014	0.003	1.037	0.026	< 0.001
P20	1.013	0.005	1.031	0.014	< 0.001
P21	1.014	0.006	1.032	0.014	< 0.001
P22	1.014	0.006	1.032	0.020	< 0.001
P23	1.015	0.006	1.034	0.020	0.001
P24	1.014	0.006	1.033	0.024	< 0.001
P25	1.015	0.005	1.035	0.023	< 0.001
P26	1.015	0.006	1.034	0.013	< 0.001
P28	1.014	0.005	1.030	0.025	< 0.001
P42	1.013	0.006	1.030	0.017	< 0.001
P56	1.016	0.005	1.031	0.010	< 0.001

Supplementary Table 3: Cumulative tortuosity index measurements in normoxic and oxygen-induced retinopathy mice. Cumulative tortuosity index (CTI) values are presented as mean ± SD for NOX and OIR mice at each postnatal age (P10–P56). CTI was computed using the iROP-Assist algorithm on vessel segmentation maps created by a Generative adversarial Network. Two-sided Mann–Whitney U tests were used to compare groups at each time point. CTI increases during the vaso-obliterative phase (P12), peaks concurrently with maximal neovascularization at P17, and remains significantly elevated relative to normoxic controls through the latest time points analyzed (P56), suggesting increased vascular tortuosity represents a durable phenotype that persists after regression of neovascular pathology.

Intervention Date	NOX Retinal Images	OIR Retinal Images
P10	8	17
P12	12	32
P13	19	21
P14	18	20
P15	12	19
P16	12	20
P17	37	23
P18	14	12
P19	10	25
P20	10	28
P21	19	22
P22	12	19
P23	13	22
P24	12	23
P25	34	14
P26	10	24
P28	15	22
P42	18	23
P56	11	29

Supplementary Table 4: Sample size of retinal flat mount images analyzed at each postnatal age in normoxic and oxygen-induced retinopathy cohorts. The number of retinal flat mount images included in quantitative analyses is reported for NOX and OIR mice at each postnatal age (P10–P 56). Counts reflect images that passed quality control for staining, flat-mount integrity, and accuracy of automated vessel segmentation prior to inclusion in neovascularization, vaso-obliteration, and tortuosity analyses.

Intervention Date	NOX Images Laboratory 1	NOX Images Laboratory 2	OIR Images Laboratory 1	OIR Images Laboratory 2
P12			24	8
P14			15	5
P15			15	4
P16			8	12
P17	28	9	4	19
P20			16	12
P23	5	8		
P42			14	9

Supplementary Table 5. Sample size distribution between two independent laboratories for retinal flat mount images analyzed at each postnatal age in normoxic and oxygen-induced retinopathy cohorts. The number of retinal flat mount images included in quantitative analyses is reported for NOX and OIR mice at each postnatal age (P10–P 56) stratified by laboratory. Counts reflect images that passed quality control for staining, flat-mount integrity, and accuracy of automated vessel segmentation prior to inclusion in neovascularization, vaso-obliteration, and tortuosity analyses.

Outcome	Main lab effect (p)	Main lab effect (β, 95% CI)	Lab-by-Age interaction (p)
NV	0.433	β = −0.0037 [−0.0128, 0.0055]	<0.001
VO	0.584	β = 0.0060 [−0.0155, 0.0275]	<0.001
CTI	0.768	β = 0.0011 [−0.0064, 0.0087]	0.484

Supplementary Table 6: **Assessment of laboratory batch effects on NV, VO, and CTI.** Using linear models, effects of systemic differences between laboratories after adjusting for age (categorical) and condition (OIR vs NOX) were analyzed. Lab effect (β, 95% CI) represents the adjusted mean difference (L2 – L1). The Lab-by-Age interaction tests whether laboratory-associated differences vary across ages.

	CTI vs NV		CTI vs VO	
**Age (P)**	**r**	**p**	**r**	**p**
10	-0.37	0.139	0.05	0.837
12	-0.05	0.784	0.02	0.926
13	-0.08	0.717	0.08	0.739
14	-0.37	0.114	0.15	0.529
15	0.02	0.949	-0.42	0.070
16	0.16	0.490	-0.47	**0.037***
17	0.42	**0.049***	0.13	0.542
18	-0.04	0.914	-0.28	0.379
19	0.22	0.303	0.35	0.084
20	-0.1	0.620	-0.32	0.092
21	0.15	0.515	-0.09	0.683
22	0.2	0.411	0.23	0.349
23	0.05	0.843	0.04	0.863
24	-0.18	0.401	-0.14	0.523
25	0.4	0.160	0.23	0.422
26	0.22	0.312	-0.11	0.613
28	-0.15	0.502	-0.26	0.247
42	0.37	0.083	-0.18	0.401
56	0.52	**0.004***	-0.25	0.197

Supplementary Table 7: Age-related relationships between CTI versus NV, and CTI versus VO in OIR mice. Spearman rank correlation coefficients (r) and corresponding p values are shown for the relationships between CTI and NV, and between CTI and VO, at each postnatal age in OIR mice. Statistically significant associations ($p < 0.05$) are indicated.

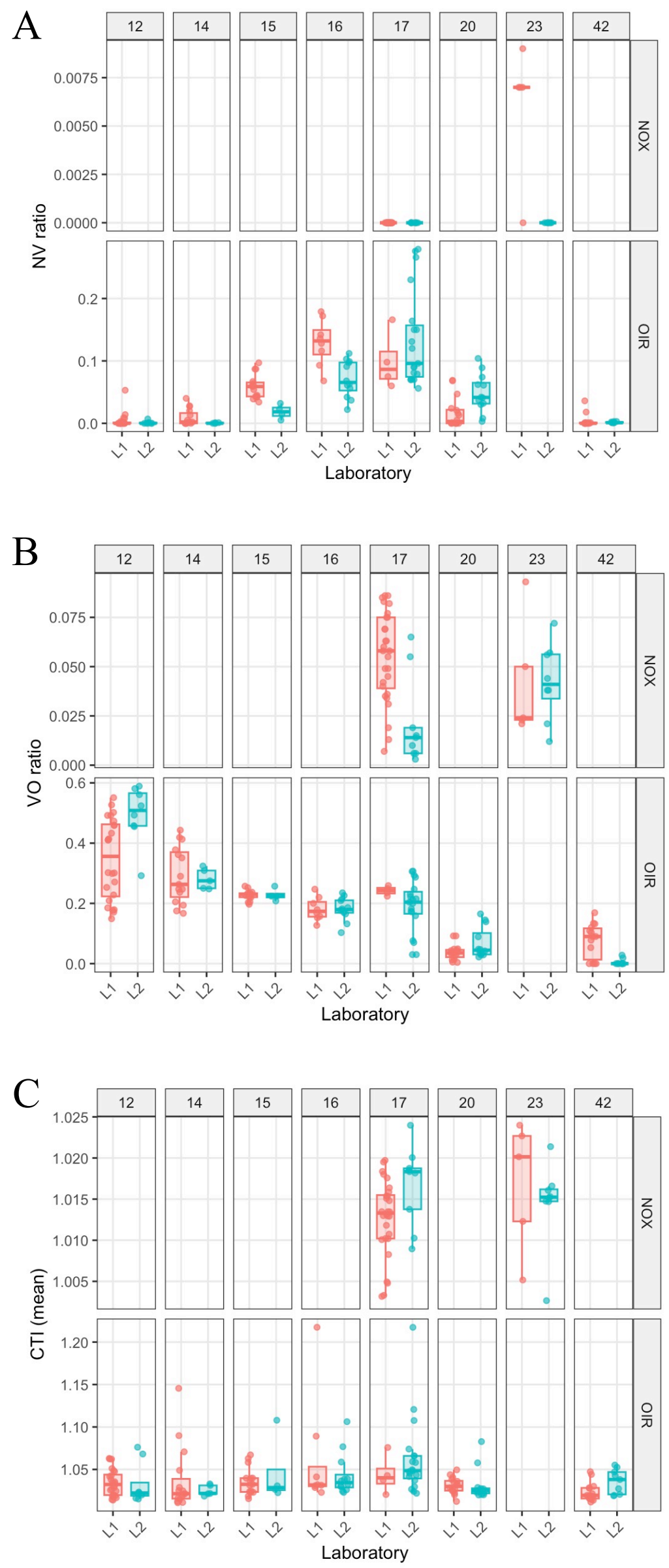

Supplementary Figure 1. Laboratory-specific batch effects of NV, VO, and CIT at various ages in NOX and OIR mice. Box-and-whisker plots represent (A) NV, (B) VO, and (C) CTI measured from retinal flat mounts obtained from NOX control (top row) and OIR (bottom row) mice across postnatal ages where preparation of mice were divvied between two laboratories. Data are stratified by laboratory of origin (L1 vs. L2) to assess batch effects. Each point represents an individual retina; boxes indicate the interquartile range, center lines denote medians, and whiskers extend to 1.5× the interquartile range.
